# General practitioners’ well-being in Belgium: results from the cross-sectional PRICOV-19 study

**DOI:** 10.1186/s12875-024-02341-4

**Published:** 2024-04-09

**Authors:** Joanna Cholewa, Cecile Ponsar, Ségolène de Rouffignac, Benoit Pétré, Esther Van Poel, Sara Willems, Michel De Jonghe

**Affiliations:** 1 Academic Center of General Medicine, Faculty of Medicine and Dentistry, UCLouvain, Brussels, Belgium; 2 Department of Public Health Sciences, Liège University, Liège, Belgium; 3 Department of Public Health and Primary Care, Ghent University, Ghent, Belgium; 4 Quality and Safety Ghent, Department of Public Health and Primary Care, Ghent University, Ghent, Belgium

**Keywords:** General practice, Primary care, Quality of care, Distress, Well-being, COVID-19, PRICOV-19, Belgium, Regions

## Abstract

**Background:**

The mental health and well-being of GPs is a critical issue as they play a vital role in providing healthcare services to individuals and communities. Research has shown that GPs often face high levels of stress, burnout, and mental health problems due to their demanding work environment. During the COVID-19 pandemic, GPs faced additional challenges which further impacted their mental health and well-being. This study aims to investigate the impact of systemic work-related stressors on the level of well-being of GPs in Belgium during the pandemic, with a particular emphasis on identifying regional variations between Flanders, Wallonia, and Brussels-Capital.

**Methods:**

Data were collected with a self-reported online questionnaire from 479 GPs Belgian practices between December 2020 and August 2021 as part of the international PRICOV-19 study that explored the organization of general practices during COVID-19 in 38 countries to guarantee safe, effective, patient-centered, and equitable care. Well-being was evaluated by the Mayo Clinic's expanded 9-item well-being index.

**Results:**

The findings of this study reveal notable regional discrepancies in the degree of well-being experienced by Belgian GPs, with the Walloon region displaying the lowest level of well-being (37%) in a population highly susceptible to professional distress (57%). Among the key stressors contributing to such distress, financial difficulties among patients (*p* < 0.011), the fee-for-service payment system (*p* = 0.013), a lack of work-related purpose (*p* = 0.047), and inadequate work-life balance (*p* < 0.001) were identified as significant factors. When examining the influence of regional disparities, it was found that the sole significant interaction between work-related stressors and region regarding the probability of experiencing distress was related to the possibility of workload sharing among practice personnel.

**Conclusion:**

The findings from this study underscore the imperative for more comprehensive research aimed at scrutinizing the differences in well-being across the three regions in Belgium and identifying the systemic factors that influence the practice environment, as opposed to exclusively concentrating on enhancing individual resilience.

## Background

The COVID-19 pandemic has precipitated an unparalleled global health crisis, severely challenging the resilience of health systems and professionals worldwide. In the face of this crisis, healthcare workers have been exposed to heightened risks of infection, increased psychological stress due to potential virus transmission to family and patients, and a significant surge in workloads [1]. These challenges have amplified the risk of burnout among healthcare professionals [1–5], including General Practitioners (GPs) – a group already vulnerable to burnout before the pandemic [2, 5, 6].

The pandemic has not only stressed the healthcare system but also necessitated a rapid and innovative reorganization of primary healthcare services [7, 8]. GPs, as key primary care providers, found themselves at the forefront of this transformative period [9, 10]. They navigated a complex landscape, simultaneously addressing acute COVID-19 cases and regular healthcare duties [11–14]. They rapidly adapted to evolving patient needs, integrated telemedicine, and upheld strict infection control standards [11–15]. Additionally, GPs constantly updated their practices based on changing guidelines [11] and served as key informers for patients about evolving public health directives [16].

Although many countries faced common challenges during the COVID-19 pandemic [17], the crisis also highlighted the influence of regional factors, including demographics, socio-economic disparities, and healthcare systems [18], particularly in federal states such as Belgium [19–22]. Belgium's healthcare governance, with responsibilities split between federal and federated authorities, provides a unique case study of this dynamic interplay [20–22]. In this structure, the regional governments of Wallonia, Brussels-Capital, and Flanders are responsible for managing primary healthcare. However, in response to the pandemic, the federal government adopted a more centralized approach, focusing on the development of comprehensive guidelines, such as social distancing, mask-wearing, testing protocols, and COVID-19 vaccine procurement and distribution strategies. Yet, the implementation of these guidelines and strategies varied greatly from region to region, leading to differences in their effectiveness [21, 23, 24]. By 2021, at the time of the survey, Belgium had experienced three waves of COVID-19 and was in its initial vaccination phase [25, 26]. During the resurgence of cases in early 2021, social distancing measures were implemented divergently across regions, leading to disparities such as varying curfew hours in Brussels-Capital, Flanders, and Wallonia [21, 23, 24]. In terms of vaccination, Brussels-Capital had the lowest rate, with 78% of adults fully vaccinated, far behind Flanders (93%) and Wallonia (88%) [26]. This situation necessitated the implementation of customized local vaccination programs, considering the unique urban and multicultural characteristics of Brussels, which differ from the broader regional landscapes of Flanders and Wallonia, encompassing both urban centers and rural areas [20, 21]. Another noteworthy example is the consistently higher COVID-19 standardized mortality rate throughout the crisis in Brussels-Capital [25]. This multifaceted landscape of regional differences in healthcare responses underlines the imperative of incorporating regional differences when assessing the impact of public health crises [20, 21].

Despite the significance of these regional variations, no studies have compared the well-being of GPs across Belgium's three regions, especially considering the substantial changes in healthcare organizations. Furthermore, in Belgium, few studies have explored the impact of the pandemic on the mental health and well-being of GPs, who serve as foundational providers of patient-centered healthcare services. This study aims to investigate the impact of systemic work-related stressors on the level of well-being of GPs in Belgium during the pandemic, with a particular emphasis on identifying regional variations between Flanders, Wallonia, and Brussels-Capital.

## Material and method

### Study design and setting

This study is part of the PRICOV-19 project, a multi-country cross-sectional study headed by Ghent University, Belgium [27]. PRICOV-19 aims to evaluate to comprehensively evaluate the organization of primary care focusing on the continuity of high-quality care, changes in task roles, the impact on the well-being of health workers, and variations across different practice types and health systems. The study design and data handling protocol are described in the Data Management Plan registered at Ghent University [27]. Data were collected in 37 European countries and Israel. In Belgium, the study was inclusive of all three regions: the Dutch-speaking Flemish Region (FL), the bilingual Brussels-Capital Region (BR), and the French-speaking Walloon Region (WL).

### Questionnaire development and data collection

Data collection was conducted through an online self-reported questionnaire among GP practices using the Research Electronic Data Capture platform (REDCap), which facilitated questionnaire hosting, distribution of invitations, and secure storage of participant responses [28]. The questionnaire was initially developed in English at Ghent University and included a pilot study with 159 GP practices in Flanders, Belgium. To address Belgium's multilingual context, it was translated into French and Dutch using a validated forward–backward method, ensuring the translations faithfully captured the nuances of the original English version, including the eWBI questions [27].

The questionnaire studied fifty-three items divided into six categories: (a) infection prevention; (b) patient flow for COVID-19 and non-COVID19 care; (c) dealing with new knowledge and protocols; (d) communication with patients; (e) collaboration within the practice and outside the practice; (f) characteristics of the respondent and the practice; and (g) well-being of the respondent. Well-being was evaluated by the Mayo Clinic expanded 9-item Well-being Index (eWBI).

### Well-being assessment using the eWBI

The eWBI (Table 1) assesses distress across various dimensions including fatigue, feelings of burnout, low overall quality of life, suicidal ideation, meaning at work, and work-life balance. Participants respond to seven items with 'yes' (assigned 1 point) or 'no' (0 points). The remaining two items use a 7-point or 5-point Likert scale. For these, selecting 'strongly disagree' or 'disagree' adds a point, while 'agree' or 'strongly agree' subtracts a point. Neutral responses do not affect the score. A cumulative score of 2 or more indicates a risk of distress. This threshold is based on empirical evidence and statistical analysis, showing a significant correlation between specific score levels and increased risk of distress or mental health issues [29].

**Table 1:** Elements of the Mayo Clinic expanded 9-items Well-being Index (eWBI)

1. During the past month, have you felt burned out from your work? Yes/No
2. During the past month, have you worried that your work is hardening you? Yes/No
3. During the past month, have you often been bothered by feeling down, depressed, or hopeless? Yes/No
4. During the past month, have you fallen asleep while sitting inactive in a public place? Yes/No
5. During the past month, have you felt that all the things you had to do were piling up so high that you could not overcome them? Yes/No
6. During the past month, have you been bothered by emotional problems (such as feeling anxious, depressed, or irritable)? Yes/No
7. During the past month, has your physical health interfered with your ability to do your daily work at home and/or away from home? Yes/No
8. The work I do is meaningful to me. From 1 (strongly disagree) to 7 (strongly agree)
9. My work schedule leaves me enough time for my personal/family life. From 1 (strongly disagree) to 7 (strongly agree)

### GP sampling and recruitment process

GPs were selected using a predefined recruitment procedure, with a preference for random selection according to the PRICOV-19 protocol [27]. A random sample of 1,477 Belgian GP practices was chosen from an updated listing on the INAMI/RIZIV web application, which included active GPs as of November 2020 [30]. Exclusion criteria for GPs included having qualified before 1980, being retired, or having low activity. Only one survey was conducted per GP practice. All selected GPs were contacted by phone, following a standardized procedure, to review their eligibility criteria and invite them to participate. This initial contact resulted in the participation of 370 GPs (participation rate of 25.1%). In addition, a convenience sample of 134 GPs was included through the networks of the research teams involved in the study. The total participation for the convenience sample study was 109 (participation rate of 81.3%). Data collection occurred between December 2020 and August 2021.

### Data analysis and variables selection

**Fig. 1:**
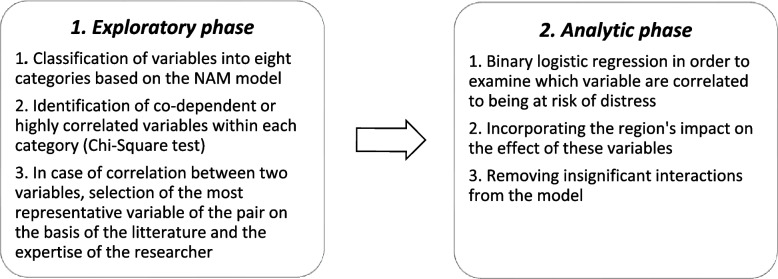
Selection of the variables to be analyzed through a stepwise approach

Statistical analysis was performed on Belgian data using SPSS software version 27.0 for Windows (IBM Corp., Armonk, N.Y., USA). Data were analyzed using a stepwise approach (Fig. 1). Initially, the fifty-three items, serving as predictor variables, were classified into eight categories: practice population profile, patient's follow-up, workload management, adjustment in practice, practice characteristics, health care role, personal factors, and regulatory environment. This categorization was inspired from the National Academy of Medicine (NAM) conceptual model of factors affecting clinician well-being [31].

The second step identified and addressed potential multicollinearity. This involved examining the variables for co-dependence or high correlation, using the Chi-Square test to assess potential correlations within each category.

The third step involved selecting the most representative variable from correlated pairs. This selection was based on empirical evidence in the literature and the expertise of our research team, ensuring the most pertinent variables were included in our analysis.

Twenty-two independent variables (Table 2) out of fifty-three variables were selected for the binary regression analysis. The covariates scored on a Likert scale were continuous covariates and the others were categorical covariates.

**Table 2:** Classification of independent predicting variables

*Item category*	*Categorical covariates*	*Continuous covariates*
Practice characteristics	Region Location of the practice Main payment system Multidisciplinary practice GP training practice	Enough protected time to review guidelines and scientific literature since the pandemic
Adjustment in practice	Telephone triage Performing video consultation Structural changes to reception area	/
Practice population profile	Number of patients with chronic conditions compared to the average PC practice Number of patients with financial problems compared to the average PC practice	/
Patient's follow-up	Contact of patients with previous problems of family violence or with a problematic child-rearing situation Contact of psychologically vulnerable patients	Contact of patients that might postpone healthcare
Workload management	/	Workload distribution between staff members in the practice Promotion of cooperation with other PC practices in the neighbourhood
Regulatory environment	/	Threat imposed by government guidelines to good practice organization Adequate government support for proper functioning of the practice
Health care role	/	Increased responsibilities since the pandemic Further training needed for amended responsibilities Meaningfulness of work
Personal factors	/	My work schedule leaves enough time for family/personal time

These twenty-two independent predicting variables were included in the binary logistic regression independently of the category in which they were classified. The outcome variable in this study is the risk of being in distress, which is determined by a cumulative score of 2 or more on the 9-item Mayo Clinic (eWBI score ≥ 2.0) [29].

A binary logistic regression was performed to examine which variables were correlated to being at risk for distress. To explore the possible impact of the region on the effect of the predictor variables, potential interactions were incorporated into the regression and interactions were removed from the model one at a time (Table 3). Non-significant interactions were eliminated, so that any interaction terms making a statistically significant contribution to the interpretation of our model was identified. The results were considered significant at the 5% uncertainty level.

**Table 3:** Items selected for the study of their interaction with the region on the effect of being at risk of distress (eWBI ≥ 2.00)

- Number of patients with chronic conditions treated compared to the average PC practice
- Workload distribution between staff members in the practice
- Promotion of cooperation with other PC practices in the neighborhood
- Increased responsibilities
- Meaningfulness of work
- Further training needed for amended responsibilities
- Work schedule leaves enough time for family/personal time

### Ethical approval

The study was conducted according to the guidelines of the Declaration of Helsinki. The Research Ethics Committee of Ghent University Hospital approved the protocol of the PRICOV-19 study (BC-07617). All participants gave their informed consent online.

## Results

### Description of the respondent sample

The number of respondents, 479 practices, was distributed among the regions as follows: 47 in BR (9.8%), 280 in FL (58.5%) and 152 in WL (31.7%) (Table 4). About two-fifths of the respondents (37.3%) were working in solo practices. In FL, the practices were more often duo or group practices compared to WL and BR (*p* < 0.001). GPs working in multidisciplinary practices were significantly higher in FL than in WL or BR (*p* = 0.016). Most of the respondent practices worked on a fee-for-service basis (91.0%). The payment system was not significantly different between the regions (*p* = 0.066). Regarding territorial location, 52.0% of the respondents were based in city-suburbs, 39.6% in mixed urban–rural areas, and only 8.4% in rural areas. Regarding the professional experience of GPs, 38.9% had less than 10 years of experience, 12.4% had 10 to 19 years of experience, 17.2% had 20 to 29 years of experience, and 31.3% had over 30 years of experience.

**Table 4:** Characteristics of the respondents' practices (*n* = 479)

Item	Characteristics	Total	BR	WL	FL	*P*-value
Location	Urban Suburban Urban–rural mix Rural	42.8% 9.2% 39.6% 8.4%	100% 0.00% 0.00% 0.00%	34.2% 11.8% 41.4% 12.5%	37.8% 9.4% 45.3% 7.6%	< 0.001
Size	Solo Duo Group (> 2)	37.3% 19.5% 43.2%	52.2% 13.0% 34.2%	58.7% 18.7% 22.7%	23.2% 21.1% 55.7%	< 0.001
Multidisciplinarity	Yes No	31.2% 68.8%	29.8% 70.2%	22.2% 77.8%	35.9% 64.1%	0.016
Main payment system	Fee-for-service Capitation fee	91.0% 9.0%	83.0% 17.0%	94.0% 6.0%	90.7% 9.3%	0.066
Work experience	0–4 years 5–9 years 10–14 years 15–19 years 20–24 years 25–29 years 30–34 years ≥ 35 years	27.0% 11.9% 5.4% 7.0% 7.7% 9.5% 11.3% 20.3%	20.9% 9.3% 4.7% 9.3% 7.0% 7.0% 11.6% 30.2%	23.3% 7.5% 6.2% 4.1% 3.4% 12.3% 13.7% 29.5%	30.3% 15.0% 5.1% 8.3% 9.8% 8.3% 9.8% 13.4%	0.004

**Fig. 2:**
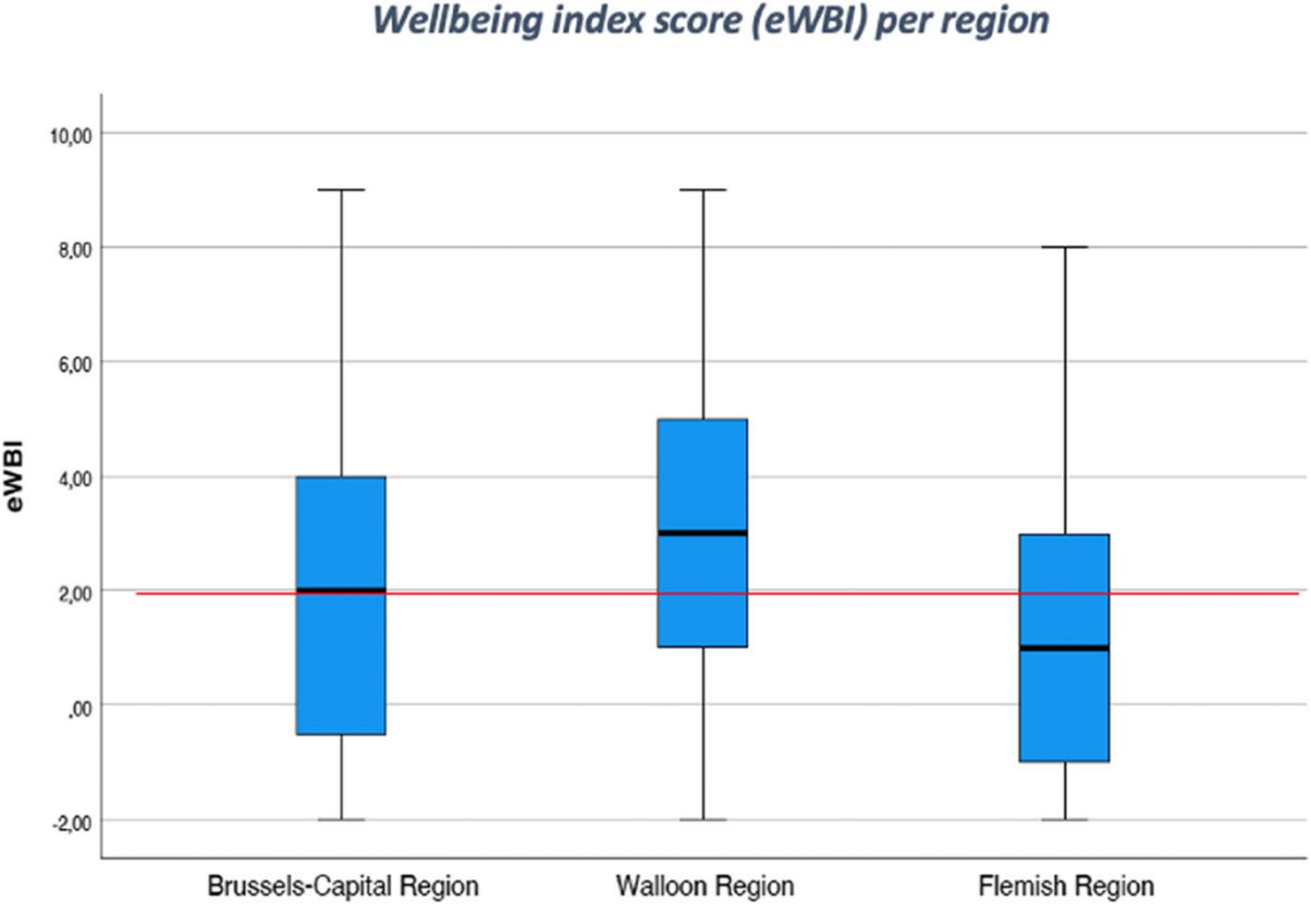
Box plot of GPs’ total eWBI scores (on a scale from -2 to 9) per region (*n* = 479)

The eWBI reveals differences in well-being across regions, as illustrated in Fig. 2. While over half of the respondents (57.0%) were identified as being at risk of mental distress in the total Belgian sample, a significant difference was observed between regions. Specifically, the WL region reported a substantially higher rate of distress (72.9%) compared to the FL and BR regions, as indicated by the statistical significance (*p* < 0.001) presented in Table 5.

**Table 5:** Regional distribution of GPs considered at risk of distress (*n* = 479)

**eWBI**	**Criteria**	**Total**	**BR**	**WL**		**FL**	***P*-value**
< 2.00	Not at risk of distress	43.0%	45.5%	27.1%		51.4%	< 0.001
≥ 2.00	At risk of distress	57.0%	54.5%	72.9%		48.6%	

*eWBI* Mayo Clinic expanded 9-items Well-being Index, *BR* Brussels Capital region, *WL* Walloon region, *FL* Flanders region

### Factors predicting the level of well-being

Regarding the factors related to the level of well-being, none of the factors of the categories of workload management, regulatory environment, patients' follow-up, and adjustments in practice had a significant impact on the eWBI of Belgian GPs after controlling for all other variables in the binary logistic regression model. However, some of the items in the four other categories significantly influenced the risk of being at distress (Table 6).

**Table 6:** Analysis of work-related factors influencing distress risk in GPs (*n* = 479)

**Work-related Factors**		**Risk of distress (eWBI** $\ge$**2.00)**		
		Estimated coefficient	*P*-value	OR
**Practice characteristics**				
Location	Urban	-0,580	0,400	0,560
	Suburban	-1,233	0,176	0,291
	Urban–rural mix	-0,379	0,560	0,685
	Rural	/	/	/
Region	WL	0,871	0,340	2,390
	BR	4,380	0,016*	79,822
	FL	/	/	/
Multidisciplinary	No	-0,546	0,251	0,580
	Yes	/	/	/
Main payment system	Fee-for-service	2,049	0,013*	7,762
	Capitation	/	/	/
Since COVID-19, there is enough protected time to review guidelines and scientific literature	a	-0,189	0,208	0,828
**Adjustments in practice**				
Does the practice perform telephone triage?	No	0,001	0,998	1,001
	Yes	/	/	/
Has the practice introduced structural changes to the reception area?	No	-0,127	0,754	0,881
	Yes	/	/	/
Does the practice perform video consultations?	No	-0,008	0,989	0,992
	Yes	/	/	/
**Practice population profile**				
GP’s perception of treating patients with chronic conditions compared to the average PC practice is:	< Average = Average > Average	0,476 -0,069 /	0,551 0,874 /	1,609 0,934 /
GP’s perception of treating patients with financial problems compared to the average PC practice is:	< Average = Average > Average	-1,720 -1,462 /	0,011* 0,010* /	0,179 0,232 /
**Patients follow-up**				
The practice actively reaches out to psychologically vulnerable patients	No	-0,559	0,236	0,572
	Yes	/	/	/
The practice actively reaches out to patients with previous problems of domestic violence or with a problematic child-rearing situation	No	-0,918	0,162	0,399
	Yes	/	/	/
The practice actively reaches out patients that might postpone healthcare	No	-0,331	0,102	0,718
	Yes	/	/	/
**Workload management**				
If staff members are absent because of COVID-19, this practice can count on the help of other staff members in the practice	a	-0,109	0,67	0,897
If staff members are absent because of COVID-19, this practice can count on cooperation with other PC practices in the neighborhood	a	-0,136	0,453	0,873
**Regulatory environment**				
Government guidelines is a threat to good practice organization	a	0,314	0,111	1,369
The government provides adequate support for the proper functioning of the practice	a	0,034	0,853	1,034
**Healthcare role**				
Since COVID-19, my responsibilities in the practice have increased	a	0,395	0,054	1,485
I need further training for amended responsibilities	a	0,256	0,182	1,291
The work I do is meaningfulness to me	b	-0,278	0,047**	0,757
**Personal and professional balance**				
My work schedule leaves me enough time for my family/personal life	a	-0,963	< 0,001**	0,382

*eWBI* Mayo Clinic expanded 9-item Well-being Index, *OR* Odds ratio

a From “I strongly disagree” to “I strongly agree” on a Likert scale of 5, b From “I strongly disagree” to “I strongly agree” on a Likert scale of 7, * The variable is a significant predicting factor of risk of distress, ** The variable is a significant protector against the risk of distress

#### Practice characteristics

Practice location (urban, suburban, rural, and mixed urban–rural) was not a factor that had a significant impact on eWBI. However, the region of the practice had an impact: a GP in BR had a higher probability of being at risk than a GP in FL (*p* = 0.047), while there was no significant difference in the probability of being at risk of distress between WL and FL. The payment system was also identified as a significant stressor. GPs working in a fee-for-service system had a higher probability of being at risk than those working in a capitation system. Multidisciplinary practice, being a training, and having sufficient protected time to review the new guidelines were not associated with a significant impact on the likelihood of being at risk for distress.

### Practice population profile

Compared to the GPs who had the perception of treating a greater number of patients with financial problems compared to the average PC practice, those who had not this impression and treated an "equal" or "lower" number of patients with financial problems compared to the average PC practice had a lower probability (*p* = 0.011) of being at risk of distress (all other things being equal). There was no significant difference regarding the perception of treating patients with a chronic condition compared to the average PC practice.

#### Healthcare role

When GPs had to face increased responsibilities during the pandemic, this did not increase the probability of being at risk for distress (*p* = 0.056). The same observation applies to the need for further training for coping with amended responsibilities (*p* = 0.178). Nevertheless, high meaningfulness of work seemed to decrease the probability of being at risk for distress (*p* = 0.047).

#### Personal and professional life balance

A work schedule leaving enough time for family/personal time significantly decreased the probability of being at risk for distress (*p* < 0.001).

#### Region

The only significant interaction between work-stress-related factors and the region on the probability of being at risk of distress was the workload distribution between staff members in the practice. This effect was significantly lower in BR than in FL (*p* = 0.006), while there was no significant difference between WL and FL.

## Discussion

The primary aim of this study was to investigate the impact of systemic work-related stressors on the well-being of GPs in Belgium during the pandemic, with a particular focus on identifying regional variations between Flanders, Wallonia, and Brussels-Capital. This research addresses a significant gap, given the scarcity of studies examining the pandemic's impact on the mental health of GPs in Belgium, especially against the backdrop of substantial changes in healthcare organizations.

The expanded 9-item Well-being Index (eWBI) was employed to assess well-being and distress, utilizing a specific score threshold (eWBI score ≥ 2.0) to identify GPs at risk of distress. This comprehensive approach aimed to cover various aspects of well-being, such as severe fatigue, suicidal ideation, feelings of burnout, quality of life, meaning at work, and work-life. By using the eWBI, the study provided a broader perspective on the well-being of GPs during the pandemic, moving beyond traditional measures like burnout alone. The use of the aggregate score (eWBI score ≥ 2.0) offered a holistic view of well-being, enabling standardized evaluation and cross-regional comparisons of individuals or populations [29, 32].

According to the study's findings, 57% of the Belgian GPs were considered at risk of professional distress during the period of December 2020 to August 2021. These results align with the PRICOV-19 study, indicating that 64% of European GPs were at risk of professional distress during the same period [32]. The study also showed that GPs with less experience, working in smaller practices, and treating more vulnerable populations, especially those caring for patients with financial difficulties, were particularly susceptible to distress [32].

In Belgium, key occupational factors negatively influencing GPs’ well-being included having more than average number of patients with financial problems, working on a fee-for-service basis compared to capitation. In contrast, finding meaning in work and having a good work-life balance were protective factors against overall distress. In 2021, the digital divide in Belgium was around 39%, a figure above the European average (34%), with only 53% of low-income households owning a laptop [33]. During the pandemic, the computerization of health care with increased use of tele- and video-consultations has thus excluded some patients from care, and those with a financial precarious background were particularly hit [34]. The difficulty in accessing care has made it more difficult for GPs to manage patients with the low income [35], a population showing a 38% higher use of GP homecare services compared to the highest income group before the health crisis in Belgium [33]. Several studies have also shown that GPs with a high share of patients with low incomes face more difficulties in managing their patients’ care because of their low health literacy, higher disease burden, increased health risks, and intertwined social determinants of health [36, 37]. It has also been reported that GPs working with low-income patients are exposed to a higher risk of burnout compared to GPs with a higher share of patients with high incomes [35–37]. The financial aspect of a GP’s patient base affects not only the health of the patient but also the well-being of the caregiver.

Among the other practice characteristics with an impact on Belgian GPs’ well-being was the payment system. Our data showed that Belgian GPs who are paid retrospectively based on activity with a fee-for-service system were at higher risk of distress compared to GPs who are paid prospectively with the capitation system. This could be explained by the fact that, during the pandemic waves, most of the consultations were done remotely. In March 2020, around 70% of the consultations were done by teleconsultations in Belgium [38]. In several countries, it has been shown that the increase in teleconsultations was correlated with a decrease in GPs’ incomes especially in the GPs’ population working with the fee-for-service system [34, 39, 40]. Furthermore, when patients came back to practice, consultation required increased treatment preparedness leading to a decrease in the number of consultations and thus of income, and increased material expenditures, such as reorganizing practice, securing supplies, and buying protective materials, leading to additional spending [40]. In this study, GPs’ sample was mainly composed of GPs working on a fee-for-service-basis (91%). This could have had an impact on the data. However, our sample of GPs reflects the situation of physicians in Belgium, as GPs are mainly self-employed and paid for fee-for-service as reported by the FPS [41]. Furthermore, some studies highlighted an increased vulnerability to the risk of burnout among GPs working on a fee-for-service regiment, well before the pandemic [42]. Also, the capitation funding is recognized as allowing more time for meetings, coordination activities, and peer-to-peer collaboration than is possible with the fee-for-service [43]. Remuneration on a fee-for-service basis appears thus as a stressor in a period of pandemic.

The two individual factors that stand out the most from this study as having a significant impact on the level of well-being of GPs are protective factors: the meaning given to work and the fact of having a good work-life balance. The notion of meaning echoes the usefulness found in the function and responsibility that the job confers [44, 45]. A study by Shanafelt found that physicians who spend less than 20% of their professional time on the activity they consider most important are three times more likely to be burnt out than those who spend at least 20% of their professional effort on this activity [46]. A recent study focusing on GP trainees during the COVID-19 crisis showed that to have meaning in work was a key factor in preventing burnout [47]. Maintaining a mentally healthy workforce to ensure quality of care and patient safety in a period of high pressure may therefore depend, among other factors, on a workforce that is able to continue to find values in their work, despite the many changes introduced by the crisis, and to maintain a good balance between their work and private life [48, 49].

When analyzing the data at the regional level, a difference in GPs' level of well-being was observed between the three Belgian regions, with more than two-thirds of Walloon respondents classified as at risk of distress, whereas, for Flemish and Brussels respondents, this proportion was 49% and 54% respectively. One explanation for this difference between regions could have been linked to the differences in primary care organization between regions. In the French-speaking part of Belgium, the primary healthcare system lacks the same level of organization seen in Flanders [23, 50]. This structural difference had notable implications during the COVID-19 pandemic. Unlike Flanders, which adopted a more structured approach with primary care zones and well-defined roles for healthcare professionals [23, 50], Wallonia struggled to coordinate its pandemic response at the local level [20, 22, 23, 50]. This resulted in scattered efforts and a heavier reliance on GPs for testing and triage, potentially causing uneven responses and less effective pandemic management in Wallonia compared to Flanders [20, 50].

When attempting to identify the effect of the region on the impact of stressors or protective factors on GPs’ well-being, the only significant interaction on the probability of being at risk for distress was the workload distribution between staff members in the practice. While several studies have shown that teamwork and group cohesion are protective factors against burnout [17, 51], the results of this study did not confirm this observation. While the Flemish GP sample had the highest percentage of group practices (55%) and the Walloon sample the lowest percentage (22%), there was no significant difference in the protective effect of workload sharing within a practice between WL and FL. In contrast, workload sharing within a practice had a weaker protective effect on GP well-being in BR compared with FL. Thus, region was not explanatory on its own. In 2011, a review of the literature had already highlighted a large disparity between French- and Flemish-speaking regions, with French-speaking GPs being more dissatisfied with their profession overall and more exposed to the risk of emotional exhaustion [52]. The low level of professional well-being of French-speaking physicians may therefore reflect a pre-existing condition that predates the Covid-19 crisis.

### Strengths and limitations

One of the strengths of the study is the use of a validated instrument for data collection.

The data shows that the GPs in our sample are distributed similarly to the overall distribution of GPs in Belgium according to the IMA-AIM Atlas 2021. For instance, in our study, 9.8% of GPs are from Brussels-Capital, 58.5% from Flanders, and 31.7% from Wallonia, which closely matches the IMA-AIM 2021 statistics that report 10.0% of GPs in Brussels-Capital, 58.0% in Flanders, and 32.0% in Wallonia [53].

Compared to the other participating countries, the Belgian response rates were slightly higher than the overall median value of 22.0% in PRICOV-19. However, some limitations should be noted. The participation to this study was entirely voluntary, which comes with a risk of self-selection bias and a rather small sample size. GP practices having a particular interest in the three main subject of this study (organization of care, quality of care and well-being during the pandemic) could have mainly participated.

Data were collected between December 2020 and Augustus 2021. This large period includes three waves of the COVID-19 pandemic in Belgium. This means that the exact time the questionnaire was answered might have affected the results and perception of the respondent towards the situation.

There are no studies in Belgium that address professional well-being in GPs and factors that might contribute to burnout. It is therefore difficult to assess whether the factors reported as having a significant effect on GPs' well-being were related to this extraordinary situation or whether they highlighted pre-existing weak links in the organisation of care.

The study's design did not allow to conduct sub-analyses based on the prevalence of COVID-19. Data collection spanned from December 2020 to August 2021, a period marked by significant changes in the COVID-19 situation. This evolving context made it challenging to perform meaningful sub-analyses at specific time points. A robust sub-analysis would require a large, consistent sample size at each time interval, a criterion difficult to fulfill given the challenges in recruiting and retaining GP participation.

## Conclusion

This study offers an insight into the well-being of GPs in Belgium during the December 2020 to August 2021 period amid the COVID-19 pandemic. It sheds light on the challenges faced by Belgian GPs, with 57% of them at risk of professional distress, aligning with trends observed in other European regions, as demonstrated in the PRICOV-19 study. These findings emphasize significant stressors, including the perception of having patient facing financial struggles and the prevailing fee-for-service payment model. The study also uncovers the protective factors contributing to the well-being of GPs, including finding meaning in their work and achieving a healthy work-life balance.

Although a significant difference in the level of well-being was observed among regions, with Wallonia displaying the highest level of GPs at risk of distress, the region alone does not fully explain this difference. This suggests that pre-existing factors may contribute to the well-being disparities that existed before the COVID-19 crisis.

These findings underscore the importance of constructive collaboration between stakeholders on the ground and relevant authorities to develop customized action plans tailored to the organizational specificities of each region in Belgium. Such a collaborative approach is crucial for improving the situation and promoting the well-being of GPs in the country.

## Data Availability

The anonymized data is held at Ghent University and all raw data that could lead to the identification of the respondents were permanently removed. Reasonable request is required to access non-identifiable data by users who are external to the research teams involved. Access will be subject to a data transfer agreement and following approval from the principal investigator of the study.

## Abbreviations

GP: General practitioner
GP practice: General practice
PRICOV-19: Quality and safety in PRImary care in times of COVid-19
BCR: Brussels-capital region
FR: Flemish region
WR: Walloon region
